# Explainable Vision Transformers and Radiomics for COVID-19 Detection in Chest X-rays

**DOI:** 10.3390/jcm11113013

**Published:** 2022-05-26

**Authors:** Mohamed Chetoui, Moulay A. Akhloufi

**Affiliations:** Perception, Robotics, and Intelligent Machines Research Group (PRIME), Department of Computer Science, Université de Moncton, Moncton, NB E1A 3E9, Canada; emc7409@umoncton.ca

**Keywords:** vision transformers, COVID-19, chest X-ray, pneumonia, radiology

## Abstract

The rapid spread of COVID-19 across the globe since its emergence has pushed many countries’ healthcare systems to the verge of collapse. To restrict the spread of the disease and lessen the ongoing cost on the healthcare system, it is critical to appropriately identify COVID-19-positive individuals and isolate them as soon as possible. The primary COVID-19 screening test, RT-PCR, although accurate and reliable, has a long turn-around time. More recently, various researchers have demonstrated the use of deep learning approaches on chest X-ray (CXR) for COVID-19 detection. However, existing Deep Convolutional Neural Network (CNN) methods fail to capture the global context due to their inherent image-specific inductive bias. In this article, we investigated the use of vision transformers (ViT) for detecting COVID-19 in Chest X-ray (CXR) images. Several ViT models were fine-tuned for the multiclass classification problem (COVID-19, Pneumonia and Normal cases). A dataset consisting of 7598 COVID-19 CXR images, 8552 CXR for healthy patients and 5674 for Pneumonia CXR were used. The obtained results achieved high performance with an Area Under Curve (AUC) of 0.99 for multi-class classification (COVID-19 vs. Other Pneumonia vs. normal). The sensitivity of the COVID-19 class achieved 0.99. We demonstrated that the obtained results outperformed comparable state-of-the-art models for detecting COVID-19 on CXR images using CNN architectures. The attention map for the proposed model showed that our model is able to efficiently identify the signs of COVID-19.

## 1. Introduction

COVID-19 refers to “Coronavirus Disease 2019”, the disease caused by a virus from the Coronaviridae family, SARS-CoV-2. This infectious disease is a zoonosis, the origin of which is still debated, which emerged in December 2019 in the city of Wuhan (Province of Hubei, China). It quickly spread, first throughout China and then abroad, causing a worldwide pandemic. COVID-19 is a respiratory disease that can be fatal in patients weakened by age or another chronic disease. It is transmitted by close contact with infected people. The disease could also be transmitted by asymptomatic patients, but there is a lack of scientific data to confirm this with certainty. Medical images such as chest X-rays (CXR) are critical to confirming a COVID-19 diagnosis, as they give physicians and radiologists visual evidence that laboratory tests are accurate. In addition, Deep Learning-based software can ease the workload of clinicians by analyzing thousands of medical images and identifying abnormalities that negate or confirm a diagnosis. AI help to detect the first cases earlier and to organize a more efficient management of healthcare personnel and medical equipment to face the peak of the pandemic. It can also make it easier to diagnose and treat the disease. Many initiatives using AI tools have also been undertaken in the health sector: some hospitals equipped themselves early on with AI-based medical imaging software to detect lung damage caused by COVID-19. Recently, Rahman et al. [1] employed deep transfer learning techniques and examined 15 pre-trained CNN models to find the best model for the COVID-19 detection. For COVID-19 CXR images, they used a COVID-19 image data collection (CIDC) dataset [2]. The other cases (Normal and Pneumonia are from RSNA dataset [3]). In their experiment, the authors used 260 CXR images for COVID-19 (70% images of each class are for training, 15% for validation). The remaining are reserved for testing. Their best model was VGG-19, which obtained the highest classification accuracy (ACC) of 89.3% with an average precision, recall, and F1-score of 0.90, 0.89, and 0.90, respectively.

Apostolopoulos et al. [4] presented a deep learning system based on MobileNet [5] and trained it from scratch for the detection of COVID-19 using CXR. The authors used 3905 CXR images from NIH dataset [6] for Pneumonia and Normal cases, and 450 CXR from the CIDR dataset for COVID-19 cases. The proposed approach achieved a classification ACC of 87.66%, 0.97 for SN, and 0.99 for SP.

Ozturk et al. [7] developed a CNN model named DarkCovidNet with 17 layers for detecting COVID-19 by using CXR images. The model was trained on 125 CXR images of COVID-19 from CIDC dataset, and the other cases are from the ChestX-ray8 dataset [8]. The authors used five-fold cross-validation and achieved an average ACC of 98.08% for binary classification (COVID-19 vs. Normal) and an ACC of 87.02%, an SN of 0.85, and an SP of 0.92 for multiclass classification (COVID-19 vs. Normal vs. Pneumonia).

Das et al. [9] used the same dataset as [7]. Their objective is to develop an automated deep transfer learning-based approach for the detection of COVID-19 infection on CXR images using Xception model [10]. The authors used 70% of the data for training, and the remaining sets are divided into 10% and 20% for validation and testing, respectively. The experimental results achieved an overall classification ACC of 97.40% with an AUC of 0.986, and the SN and SP are 0.97 and 0.97, respectively.

Iraji et al. [11] presented a hybrid approach based on deep CNN for COVID-19 detection. The feature vectors were extracted from CXR images using a deep CNN. In addition, the binary differential metaheuristic algorithm was used to select the most valuable features. Finally, an SVM classifier was given these optimized features. The authors trained and tested the algorithm on the dataset containing CXR images from three categories (364 images for each), including COVID-19, Pneumonia, and Normal, obtained from the CIDC and “Labeled optical coherence tomography (OCT) and Chest X-ray images for classification” datasets [12], with a total of 1092 CXR images. The proposed method achieved an ACC of 99.43%, an SN of 0.99, and an SP of 0.99.

Yousefi et al. [13] used a 2D U-Net [14] model to segment the lung lobes. The authors extracted deep latent space radiomics by applying a deep convolutional autoencoder with internal dense layers to extract low-dimensional deep radiomics. They used the Johnson–Lindenstrauss lemma, Laplacian scoring, and principal component analysis to reduce dimensionality in conventional radiomics. For multiclass classification, they obtained an ACC of 72.5%.

Arias-Garzn et al. [15] used popular deep learning models (VGG19 and U-Net) to classify CXR images as positive or negative COVID-19. First, the authors used U-Net [14] for lung segmentation in order to remove the surroundings which do not offer relevant information for the task. After segmentation, they used VGG19 [16] for classification. The authors developed their algorithm using BIMCV-COVID19+, BIMCV-COVID-19 and Spain Pre-COVID-19 era datasets [17]. Their system achieved an ACC of 97% for COVID-19 detection.

Recently, various studies used the Vision Transformer (ViT) models for COVID-19 detection instead of CNN architectures. Shome et al. [18] proposed a ViT for COVID-19 detection using CXR images. The authors collected data from three open-source datasets of CXR images including COVID-19, Pneumonia, and Normal cases from datasets by El-Shafai et al. [19], Sait et al. [20], and Qi et al. [21] to obtain a total of 30,000 images. The proposed ViT model achieved an ACC of 98% and an AUC score of 0.99 in the binary classification task. For the multi-class classification (COVID-19, Normal, and Pneumonia), they obtained an ACC of 92% and an AUC score of 0.98.

Mondal et al. [22] presented the use of ViT instead of CNN for COVID-19 detection using CXR and CT images. The authors employed a multi-stage transfer learning technique to address the issue of data scarcity. They constructed a custom dataset consisting of three cases: Normal, Pneumonia, and COVID-19 were obtained from the Kaggle repository [12,23]. Their algorithm achieved an ACC of 96%, 0.96 for SN, and 0.97 for SP.

Sivarama et al. [24] used pretrained ViT models for detecting the presence of COVID-19 disease on CXR images. They trained and tested their approach on 9443 CXR images for COVID-19 and 9662 CXR images for Non-COVID-19, and the CXR images were obtained from the “COVID19, Pneumonia, and Normal Chest X-ray PA” [25] and the COVID-19 X-ray datasets [26,27]. This approach achieved an ACC of 97.61%, a precision score of 95.34%, a recall score of 93.84%, and an F1-score of 94.58%.

Park et al. [28] proposed ViT by using the low-level CXR feature to extract the COVID-19 CXR features. Their backbone network was DenseNet and trained using a large public datasets to obtain the abnormal features. Then, the embedded features from the backbone network were used as the corpus for ViT training. The authors tested their method on three datasets: CNUH [29], YNU [30], and KNUH [31]. They achieved an average AUC of 0.94, 0.90, and 0.91, an average SN of 0.87, 0.85, and 0.85, an average SP of 0.91, 0.84, and 0.84, and an average ACC of 86.4%, 85.9%, and 85.2% on the CNUH, YNU, and KNUH datasets, respectively.

In this paper, we investigate the use of recent deep learning models for a multiclass classification problem to detect COVID-19, Pneumonia, and Normal cases using CXR images. The proposed models were inspired by the Transformer scaling successes in NLP named Vision Transformers (ViT). We fine-tuned and trained the models on CXR images in a supervised manner by using a large dataset of COVID-19 with 7598 CXR images obtained from the SIIM-FISABIO-RSNA COVID-19 dataset [32]. The other cases (Normal and Pneumonia) are obtained from RSNA dataset [3]. Several performance measures were used to validate the performance of our proposed ViT models in terms of Accuracy, AUC, Sensitivity, and Specificity. We aim to make the following contributions:

1. Several Vision Transformers models have been fine-tuned to test the performance of these models in the classification of CXR images in comparison with convolutional neuron networks (CNN).
2. Two large datasets and more than 20,000 CXR images are used to assess the efficiency of the proposed technique.
3. We experimentally demonstrated that our proposed approach outperforms the previous models for COVID-19, as well as other CNN and Transformer-based architectures, especially in terms of the generalization on unseen data.
4. The Attention map for the proposed models showed that our model is able to efficiently identify the signs of COVID-19.
5. The obtained results achieved a high performance with an Area Under Curve (AUC) of 0.99 for multi-class classification (COVID-19 vs. Pneumonia vs. Normal). The sensitivity of the COVID-19 class achieved 0.99.

**Figure 1:**
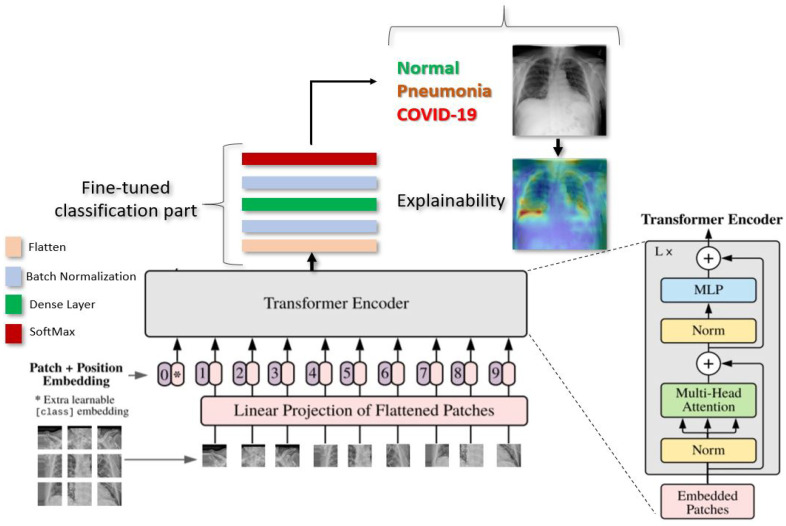
Illustration of our proposed ViT model for COVID-19 detection using CXR images. The input image is split into equal-sized patches and embedded using linear projection. Position embeddings are added, and the resulting sequence is fed to a Transformer encoder.

Figure 1 gives an overview of our proposed deep learning approach for COVID-19 detection.

## 2. Datasets

### 2.1. SIIM-FISABIO-RSNA COVID-19

**Figure 2:**
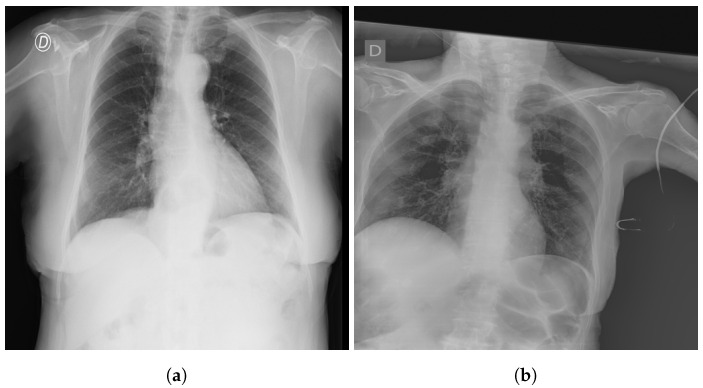
Example of SIIM-FISABIO-RSNA COVID-19 images: (**a**,**b**) [32].

The SIIM-FISABIO-RSNA COVID-19 [32] is a public dataset provided by Society for Imaging Informatics in Medicine (SIIM). Each CXR image comes with labeling to identify and localize COVID-19 abnormalities in chest radiographs. The competition is an object detection and classification problem. In our study, we used all images annotated COVID-19 for the classification. The train dataset comprises 6334 CXR images in DICOM format, which were de-identified to protect patient privacy. All images were labeled by a panel of experienced radiologists for the presence of opacities, as well as overall appearance. The test set contains 1264 CXR images. Figure 2 shows an examples of CXR from the SIIM-FISABIO-RSNA COVID-19 dataset.

### 2.2. RSNA

**Figure 3:**
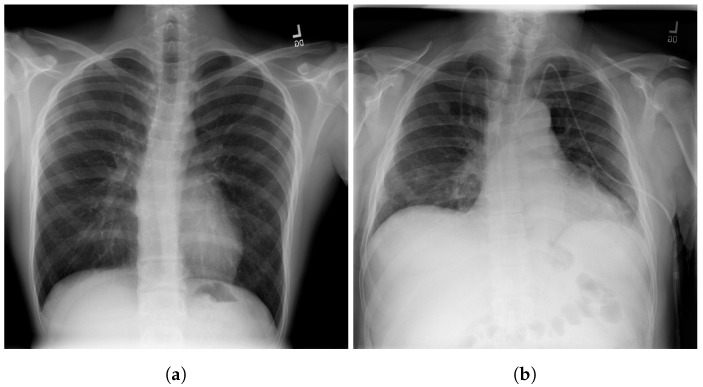
Examples of RSNA images: (**a**) Normal; (**b**) Pneumonia [3].

The RSNA dataset [3] is a public dataset of CXR images with patients’ metadata. This dataset was provided for a challenge in Kaggle by the US National Institutes of Health Clinical Center [33]. It contains 26,684 CXR images for unique patients, and each image is labeled with one of three different classes from the associated radiology reports: ’Normal’, ’No Lung Opacity/Not Normal’, and ’Lung Opacity’. Figure 3 shows images examples from the RSNA dataset.

## 3. Vision Transformer Model

In this section, we present the details of the proposed deep learning Vision Transformers model [34] for detecting COVID-19, Pneumonia, and Normal cases.

As illustrated in Figure 1, Vision Transformer model split an image into *K* small patches, with each patch containing N × N pixels. A flattened vector (2D to 1D) of pixel values from a patch of size N × N is used as the input sequence. Each flattened element is sent into a linear projection layer, which produces the “Patch embedding”. Image patches are treated the same way as tokens (words) in an NLP application.

According to the position of the image patch, an extra learnable (class) embedding is added to the sequence. After being updated by self-attention, this class embedding is utilized to predict the class of the new image.

### 3.1. Patch Embedding

The most important part of transformers is how to break down the image into patches. An image is presented as 3DImage(X)∈ resolution $R^{H\times W\times C}$ and reshapes the 3D image into flattened 2D patches as PatchImage ($X_{p}$) ∈$R^{N\times (P^{2}.C)}$, where the sequence length is $N=H.W/P^{2}$, (P,P) is the resolution of each image patch, and each patch is a *D* dimension vector with a trainable linear projection.

### 3.2. Class

Similar to BERT [35], the authors [34] prepend a learnable embedding to the sequence of embedded patches $z_{0}^{0}=X_{class}$ and $z^{0}=\left[X_{class};X_{p}^{1}E;X_{p}^{2}E;...;X_{p}^{N}E\right]+E_{pos},E\in R^{(P^{2})\times D},E_{pos}\in R^{(N+1)\times D}$. $X_{class}$ is a class label, and $X_{p}^{N}$ is patch images *N*∈ 1 to *n*.

The transformers always need a Class label at the 0th position when using the transformer encoder to pre-train. When passing patch images as inputs, one classification token must always be prepended as the first patch, as illustrated in Figure 1.

### 3.3. Positional Encodings/Embeddings

Because Transformers must learn inductive biases in order to perform the task for which they are being taught, it is always advantageous to support that learning process in any manner possible. Any inductive bias that it can introduce into the model’s inputs will help it learn faster and produce better results.

To keep positional information, position embeddings are appended to the patch embeddings. These embeddings can indicate the position of a feature in a one dimensional flattened sequence or a two dimensional position of a feature in computer vision.

### 3.4. Model Architecture

The transformer has no idea what order the images are in if we do not provide it the positioning data (which come first and the images that follow it). The transformer encoder receives this sequence of vector images. A Multi-Head Self Attention (MSA) layer and a Multi-Layer Perceptron (MLP) layer make up the Transformer encoder module. The Multi-Head Self Attention layer divides inputs into several heads, allowing each head to learn varying levels of self-attention. All of the outputs of the heads are then combined and fed into the Multi-Layer Perception.

In this work, we used three variations of Vision Transformer models, ViT-B16, ViT-B32, and ViT-L32, to detect COVID-19, Pneumonia, and Normal cases in CXR images. The objective in this study is to test the performance of these models for the classification task of COVID-19 with other cases.

We choose the ViT-B16, ViT-B32, and ViT-L32 models as the most suitable amongst those tested for further experimentation.

During pre-training, an MLP is used to represent the classification head and it is replaced by a classification part during the fine-tuning stage. As illustrated in the Figure 1. for each ViT model, we added flatten layer, followed by a Batch-Normalization and a Dense layer of size 11 followed by another layer of Batch-Normalization. Finally, the Softmax function gives the probability for the classification of CXR images into Normal, COVID-19. or Pneumonia.

## 4. Experimentation

To train ViT models for detecting COVID-19, Normal, and Pneumonia cases, we used 7598 CXR images of COVID-19, 8552 for Normal, and 5674 for Pneumonia, we obtained a total of 21,824 CXR images, including 18,561 CXR images for the models’ training and 3263 for the testing. Data augmentation was applied during training with a zoom and shear range of 0.2 and horizontal flip. We used a RectifiedAdam [36] optimizer with a fixed learning rate of $1\times 10^{-4}$ The batch size was set to 16 for ViT-B16/32 and 4 for ViT-L32. The models were trained for 200 epoch. All CXR images were resized to 512 × 512. These optimal hyperparameters were determined experimentally. We adopted AUC, ACC, SN, and SP as our evaluation metrics. Pre-processing, development, and evaluation of the algorithm was implemented using Keras [37] on an Nvidia Tesla V100 and an Nvidia RTX 2080 Ti [38].

Table 1 summarizes the hyperparameters used with our ViT models

### 4.1. Metrics

In this work, we used the following metrics: accuracy (ACC), sensitivity (SN), specificity (SP), and area under curve (AUC) [39]. The SN and SP show the performance of the proposed approach. The AUC computed using the ROC curve is a performance measure widely used for medical classification problems to highlight the compromise between good and bad classifications by the model. These metrics are defined in the following:

(1) $$SN=\frac{TP}{TP+FN}$$

(2) $$SP=\frac{TN}{TN+FP}$$

(3) $$ACC=\frac{TP+TN}{TP+FN+TN+FP}$$

where TP is the True Positive rate and represents the number of positive cases which are labeled correctly; TN is the True Negative rate and means the number of negative cases which are labeled correctly; FP is the False Positive rate, the number of positive cases which are labeled falsely; and FN is the False Negative rate for the number of negative cases which are labeled falsely.

### 4.2. Results

In this section, we present the results obtained by the proposed ViT models for multiclass classification (COVID-19, Pneumonia, and Normal). As we can see from Table 2, ViT-B32 achieved the highest performance with an average SP and SN of 0.96 and 0.96, respectively. The obtained AUC was 0.991 using 3263 CXR images in the test set. The ViT-B16 provided an interesting score with an AUC of 0.960 and an average SN and SP of 0.86 and 0.87, respectively. Still, ViT-L32 achieved the lowest score compared to the other ViT models, with an AUC of 0.7911. This shows that the larger models are not better than the smaller ones (ViT-B16, ViT-B32).

**Figure 4:**
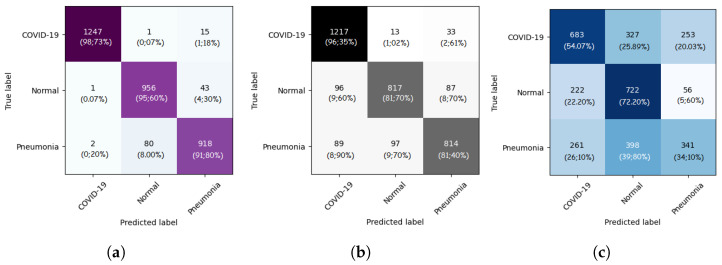
Confusion matrices of ViT-B16 (**a**), ViT-B32 (**b**), and ViT-L32 (**c**) (COVID-19 vs. Pneumonia vs. Normal) classification.

**Figure 5:**
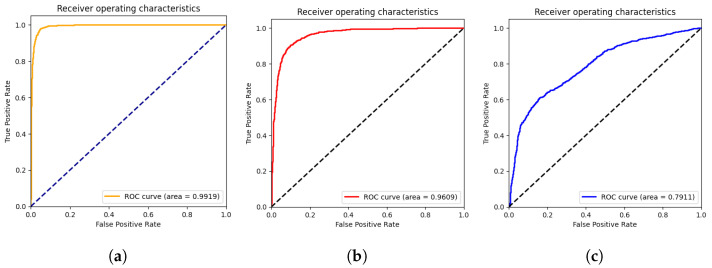
ROC curves of ViT-B16 (**a**), ViT-B32 (**b**), and ViT-L32 (**c**) (COVID-19 vs. Pneumonia vs. Normal) classification.

The confusion matrices for the ViT-B16, ViT-B32, and ViT-L32 models are given in Figure 4. As we can see, for the ViT-B32 model, only 16 CXR images from 1263 images of COVID-19 class were misclassified. For the Normal class, only 44 CXR images from 1000 were misclassified. Figure 5 show the ROC curves for the ViT-B16, ViT-B32, and ViT-L32 models. We can see the high performance achieved by the ViT-B32 model.

## 5. Model Explainabilty

**Figure 6:**
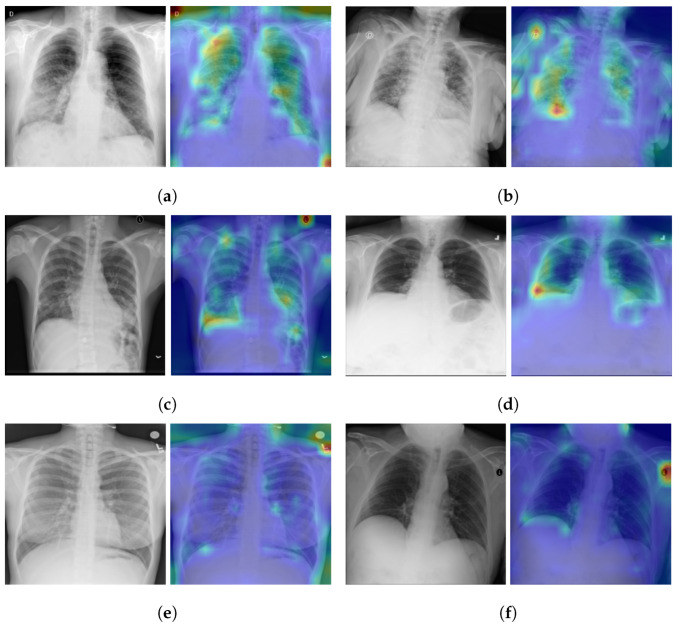
Explainabilty of TP and TN cases: (**a**,**b**) TP of COVID-19, (**c**,**d**) TP of Pneumonia, and (**e**,**f**) TN of Normal. The green and yellow/red colors highlight important areas detected by the ViT model (ViT-B32) on the CXR images by using the attention weights from transformer block.

As illustrated in the Figure 1, the vision transformer is made up of a Standard Transformer Encoder and a Self-Attention with MLP module. To understand how the model learned to detect the signs of Pneumonia pathology, including COVID-19 signs, we visualized the signs detected by ViT by using the attention map of the best model (ViT-B32). The input image can be visualized using the self-attention score for the model. Figure 6 shows samples of TPs and TNs and their attention maps. As we can see in Figure 6a,b, the attention map localizes the most important signs of COVID-19. Similarly, in Figure 6c,d, the opacity on the lungs is localized by the attention map. Figure 6e,f are the TNs, and the model does not detect any signs on the lungs for Normal images.

### Performance Comparison

To compare the performance with the other baseline CNN-based models, we fine-tuned the following CNN models: EfficientNet-B7, EfficientNet-B5, DenseNet-121 and NASNet-A-Large as the state-of-the-art (SOTA) CNN-based models. For comparison with other Transformer-based models, we used ViT-B32. All CNN models used the same pre-training process on RSNA and IIM-FISABIO-RSNA COVID-19 datasets and were subsequently trained and evaluated in the same way as the proposed ViT models for a fair comparison. As suggested in Table 3, fine-tuned ViTB-32 outperformed the CNN-based models.

The performance comparison with recent methods for COVID-19 detection using CNN architectures is presented in Table 4. We can see that the fine-tuned model (ViT-B32) outperforms most of the recently published work for COVID-19 detection using CXR images and CNN networks.

ViT-B32 obtained the same scores as Luz et al. [40] in terms of sensitivity; still, our proposed models perform best for the ACC. The work of Luz et al. [40] used only 183 COVID-19 images (vs. ours, which used 7598), which does not help in evaluating the generalization performance of the algorithm on large data.

Our results also outperform the results of the work by Wehbe et al. [41]. The authors developed their models on 4253 of COVID-19 CXR images. According to their paper, their model surpassed radiologists’ performance, which leads us to think that our model will have an even better performance if compared to a manual interpretation by radiologists.

The number of COVID-19 images used in our study is higher compared to other studies, which confirms the robustness of the proposed Vision Transformer model (ViT-B32) for detecting COVID-19.

The ViT-B32 model improves our previous work [42], which was developed using 3288 COVID-19-positive CXR images. Our previous model was based on the CNN named EfficienNet-B5 [43] and obtained an AUC of 0.97, an SP of 0.94, an SN of 0.97, and an ACC of 93%. The same architecture used in [44], which was developed using 2385 of COVID-19-positive CXR images and obtained an AUC of 0.95 [13], an SP of 0.90, and an SN of 0.97.

When compared to Transformer-based models, the proposed fine-tuned ViT-B32 model showed statistically better performance than other Transformer-based models. Table 5 shows the performance comparison of the proposed ViT-B32 model with recent published approaches using Vision Transformers’ models for COVID-19 detection. As we can see, the fine-tuned ViT-B32 model outperforms most of the recently published work for COVID-19 detection using Vision Transformers. Krishnan et al. obtained an ACC score of 97.61% because their approach is based on a binary classification, and our performance is based on the distinction between three classes (COVID-19, Pneumonia, and Normal).

These results suggest that our model offers better generalization performances in multi-class classification tasks compared with the existing model architectures.

The attention map shows a very precise localization of the COVID-19 signs and can be used as a CAD tool to further help physicians in their diagnosis.

## 6. Conclusions

In this work, we developed various models based on Vision Transformers, ViT-B16, ViT-B32, and ViT-L32, for COVID-19 detection on chest X-ray images. The Best model was ViT-B32. ViT-B32 was fine-tuned to detect COVID-19 using 7598 COVID-19-positive CXR images.

The obtained results show that the best model (ViT-B32) outperforms recent deep learning approaches for COVID-19 detection on CXR using deep CNN networks. The model achieved high AUC scores with 0.991 and a specificity and sensitivity of 0.96 and 0.96, respectively. For the COVID-19 class, we obtained a sensitivity of 0.99, a specificity of 0.99, and an accuracy 0.99, meaning that we have a better performance in measuring the proportion of actual positives that are correctly identified as such (e.g., the percentage of people who are correctly identified as having COVID-19).

The attention map for the Vision Transformer model showed that ViT-B32 is efficient in identifying the most important pathology regions (the signs of COVID-19) and other Pneumonia signs. The proposed technique is an interesting contribution in the development of a CAD system able to detect COVID-19 and other Pneumonia cases in CXR images. 

## Figures and Tables

**Table 1:** Hyperparameter Configuration.

Configuration	Value
Optimizer	RectifiedAdam
Epoch	200
Batch size	16 for ViT-B16/32 and 4 for ViT-L32
Learning rate	$1\times 10^{-4}$
Batch Normalization	True

**Table 2:** ACC, AUC, SN, and SP of our deep Vision Transformers models for detecting COVID-19, Pneumonia, and Normal.

Model	ACC %	AUC	SN	SP
ViT-B16	87.00	0.96	0.86	0.87
**ViT-B32**	**96.00**	**0.99**	**0.96**	**0.96**
ViT-L32	53.00	0.79	0.54	0.54

**Table 3:** ACC, AUC, SN, and SP of our deep Vision Trans- former models for detecting COVID-19, Pneumonia, and Normal Vs. existing SOTA CNN models.

Model	ACC (%)	AUC	SP	SN
EfficientNet-B7	93.82	0.95	0.92	0.93
EfficientNet-B5	94.64	0.95	0.83	0.92
DenseNet-121	88.13	0.90	0.91	0.87
NasNetLarge	94.48	0.96	0.90	0.96
MobileNet	93.16	0.95	0.92	0.94
**ViT-B32**	**96.00**	**0.99**	**0.96**	**0.96**

**Table 4:** Performance comparison with state-of-the-art methods using CXR images for COVID-19 detection.

Ref.	Dataset	#COVID-19 Images	ACC %	AUC	SN	SP
Rahman et al. [1]	CIDR	260	89	-	-	-
Afshar et al. [45]	Unspecified	N/A	95	0.970	0.90	0.95
Apostolopoulos et al. [4]	CIDR	450	87	-	0.97	0.99
Luz et al. [40]	CIDR	183	93	-	0.96	-
Ozturk et al. [7]	CIDR	125	87	-	0.85	0.92
Das et al. [9]	CIDR	N/A	97	0.986	0.97	0.97
Wehbe et al. [41]	Multiple institutions	4253	83	0.900	0.71	0.92
**ViT-B32**	**RSNA**	**7598**	**96**	**0.991**	**0.96**	**0.96**

**Table 5:** Performance comparison with state-of-the-art methods using Vision Transformers for COVID-19 detection.

Ref.	#CO-19 Images	ACC	AUC	SN	SP	Classification
[24]	3500	97.61	-	0.93	-	Binary-class
	12,083	92.00	0.980	-	-	Multi-class
[22]	2358	96.00	-	0.96	0.97	Multi-class
[28]	2431	86.40 95.90 85.2	0.941 (CNUH) 0.909 (YNU) 0.915 (KNUH)	0.87 (CNUH ) 0.85 (YNU) 0.85 (KNUH)	0.91 (CNUH) 0.84 (YNU) 0.84 (KNUH)	Multi-class
**ViT-B32**	**7598**	**96.00**	**0.991**	**0.96**	**0.96**	Multi-class

## Data Availability

The data used in this work come mainly from public datasets. Please see the section describing the datasets.
